# Choroidal Thickness and Hemoglobin A1c Levels in Patients with Type 2 Diabetes Mellitus

**DOI:** 10.18502/jovr.v14i3.4784

**Published:** 2019-07-18

**Authors:** Hamidreza Torabi, Mohsen Saberi Isfeedvajani, Majid Ramezani, Seyed-Hashem Daryabari

**Affiliations:** ^1^Health Management Research Center, Baqiyatallah University of Medical Sciences, Tehran, Iran; ^2^Medicine, Quran and Hadith Research Center and Department of Community Medicine, Faculty of Medicine, Baqiyatallah University of Medical Sciences, Tehran, Iran; ^3^Nephrology and Urology Research Center, Baqiyatallah University of Medical Sciences, Tehran, Iran

**Keywords:** Choroidal Thickness, Diabetes Mellitus, Diabetic Retinopathy, Enhanced Depth Imaging Optical Coherence Tomography, HbA1c

## Abstract

**Purpose:**

The aim of this study was to assess the correlation of hemoglobin A1c (HbA1c) levels with choroidal thickness in patients with type 2 diabetes mellitus (DM) using spectral domain optical coherence tomography (SD-OCT).

**Methods:**

In this prospective case series, 180 eyes from 90 patients with type 2 DM were classified into three study groups based on HbA1c values: group 1 included patients with good glycemic control (HbA1c ≤ 7%), group 2 included patients with moderate glycemic control (HbA1c between 7% and 8%), and group 3 included patients with poor glycemic control (HbA1c ≥ 8%). Additionally, 50 eyes from 25 non-diabetic subjects were enrolled to group 4 as a control group. Sub-foveal, nasal, and temporal choroidal thickness were measured and compared.

**Results:**

Mean central, nasal, and temporal choroidal thicknesses in diabetic patients (247.80, 238.63, and 239.30 μm) were significantly less than non-diabetic healthy subjects (277.56, 262.92, and 266.32 μm). Additionally, mean central, nasal, and temporal choroidal thickness values in group 4 (277.56, 262.92, and 266.32 μm) were significantly greater than the corresponding values in group 2 (248.34, 237.55, and 236.45 μm) and group 3 (239.81, 234.62, and 233.94 μm), but was not significantly different from corresponding values in group 1 (259.46, 246.12, and 251.00 μm).

**Conclusion:**

HbA1c values have a significant correlation with choroidal thickness in diabetic patients, and better glycemic control with HbA1c ≤ 7% may prevent choroidal thinning.

##  INTRODUCTION

Diabetic retinopathy (DR) is one of the major causes of visual impairment worldwide.^[1]^ The clinical features and pathogenesis of DR are primarily related to retinal vascular changes; however, choroidal vascular damage may have an important role.^[2]^ Delayed filling of the choroidal vessels using indocyanine green angiography has been reported in eyes with DR.^[3,4,5]^ Moreover, reduction in the choroidal blood flow and choroidal volume has been demonstrated in eyes with both non-proliferative and proliferative DR using Doppler flowmetry.^[6]^ Choroidal vascular changes, including choroidal aneurysms, choroidal vessel narrowing and tortuosity, choroidal neovascularization, and choroidal non-perfusion may occur in eyes with DR.^[7,8]^

Optical coherence tomography (OCT) is a non-invasive method that allows high resolution *in vivo* imaging of the posterior segment of the eye.^[9]^ Nowadays, improvement of the enhanced depth imaging (EDI) software permits highly reliable measurement of the choroidal thickness.^[10,11]^

Hemoglobin A1c (HbA1c) is one of the standard tools for the assessment of glycemic control and its optimum value is 5.6–7% in patients with diabetes.^[12]^ A previous study reported that at least 1% reduction in HbA1c can lead to significant reduction of the serious complications of DM, including death, myocardial infarction, and microcellular damage. ^[13]^

The aim of this study was to evaluate the correlation of HbA1c values with choroidal thickness in diabetic patients using EDI-OCT.

##  METHODS

Patients with type 2 diabetes mellitus (DM) with no evidence of DR or with mild non-proliferative diabetic retinopathy (NPDR) were enrolled in this cross-sectional study. The disease severity was determined based on the clinical findings and using the International Clinical Disease Severity Scale for DR.^[14]^ In eyes with "no diabetic retinopathy", as the name implies, there were no diabetic changes in the fundus examination, and eyes with "mild NPDR" were characterized only by the presence of a few microaneurysms. All fundus examinations were performed by a single vitreoretinal specialist.

The exclusion criteria were the presence of DR more than mild NPDR, diabetic macular edema (DME), any other ocular disorders, history of ocular surgery, including laser photocoagulation or intraocular anti-vascular endothelial growth factor (anti-VEGF) injection, myopia more than -3 diopters, hyperopia more than +3 diopters, and patients with a history of hypertension or any other systemic diseases besides DM.

The current study was approved by the Ethics Committee of our institute and was performed in agreement with the ethical principles of the Declaration of Helsinki. Informed consent was obtained from every enrolled subject.

EDI-OCT using Heidelberg spectral domain OCT (SD-OCT; Spectralis, Wavelength: Heidelberg Engineering Co., Heidelberg, Germany) was performed without pupillary dilation on the same day as blood sampling. Complete ophthalmic examinations were performed one or two days before EDI-OCT imaging. All OCT scans were performed by the same experienced operator between 11 and 11:45 am to avoid potential choroidal thickness changes with circadian rhythm.

Central sub-foveal choroidal thickness was measured using the caliper that is present (Spectralis software version 5.3; Heidelberg Engineering) from the hyper-reflective line corresponding to the Bruch's membrane under the retinal pigment epithelium (RPE) to the interface of the choroid and sclera, in the central horizontal B-scan passing directly through the foveal center. Then, the nasal and temporal choroidal thickness were measured 500 μm nasal and temporal to the central sub-foveal point. Choroidal thickness measurements were performed by a single vitreoretinal specialist who was masked to the identity of the patients and the study groups. Twenty-five patients were selected randomly from different groups and their choroidal thickness was measured again. Intraclass correlation coefficient for intra-observer reproducibility was 0.91. Patients with inadequate image quality in whom the interface of the choroid and sclera was not recognizable were excluded.

All subjects were enrolled into three study groups based on HbA1c values:

Group 1 included patients with good glycemic control (HbA1c ≤ 7%), group 2 included patients with moderate glycemic control (HbA1c between 7% and 8%), and group 3 included patients with poor glycemic control (HbA1c ≥ 8%). Healthy non-diabetic subjects were enrolled to group 4 as a control group. Sub-foveal, nasal, and temporal choroidal thickness were measured and compared in the eyes of all study groups.

### Statistical Analysis

Data were analyzed using SPSS version 24 (IBM Corp, Armonk, NY, USA). Mean ± SD was reported for quantitative variables. Percentage was reported for qualitative variables. The Kolmogorov-Smirnov test was used to evaluate normal distribution in quantitative variables. Analysis of variance (ANOVA) or an equivalent non-parametric test (Kruskal-Wallis H test) was used to compare quantitative variables between the four groups. Post hoc test (Tukey test) was used if the ANOVA was significant. A correlation test (Pearson coefficient when the variables had normal distribution or Spearman coefficient when the distribution of variables was not normal) was used to evaluate the correlation of choroidal thickness and quantitative variables, such as fasting blood sugar (FBS), duration of diabetes, etc. An independent sample's t-test or equivalent non-parametric test (Mann-Whitney U test) was used to compare between two groups, such as the diabetic and non-diabetic ones. The paired sample t-test was used to compare variables within each groups such as the comparison of central choroidal thickness between right and left eyes. P-value < 0.05 was considered significant.

##  RESULTS

A total of 180 eyes from 90 diabetic patients and 50 eyes from 25 non-diabetic patients were enrolled in this study. Group 1 included 48 eyes from 24 diabetic patients with good glycemic control (HbA1c ≤ 7%), group 2 included 58 eyes from 29 diabetic patients with moderate glycemic control (HbA1c between 7% and 8%), and group 3 included 74 eyes from 37 diabetic patients with poor glycemic control (HbA1c ≥ 8%). Additionally, group 4 included 50 eyes from 25 non-diabetic subjects as the control group.

The mean age and duration of DM was similar among all study groups [Table 1]. There was no significant difference between mean central choroidal thickness of the right (247.80 ± 39.74 μm) and left eyes (248.12 ± 37.40 μm) of diabetic patients in all study groups (P = 0.849).

**Table 1:** Demographic data of subjects in all study groups


	**Group1**	**Group2**	**Group3**	**Group4**	**** ***P*** **-value**
Number of cases (patients/eyes)	24/48	29/58	37/74	25/50	
Age	56.20 ± 8.13	59.02 ± 6.43	57.45 ± 7.44	59.10 ± 6.43	0.723
Sex (male/female)	13/11	16/13	17/20	13/12	0.682
Duration of DM (years)	6.12 ± 4.51	6.81 ± 5.20	7.10 ± 4.26	0.249
Mean HbA1c	6.12 ± 0.53	7.55 ± 0.39	10.21 ± 1.16	<0.001
	
	
DM, diabetes mellitus; HbA1c, hemoglobin A1c					

Overall, the mean central, nasal, and temporal choroidal thickness in diabetic patients (180 eyes) were significantly lower than those in the non-diabetic healthy subjects (50 eyes) [Table 2]; however, the mean central macular thickness in the diabetic eyes (251.51 ± 26.22 μm) and non-diabetic eyes (246.24 ± 22.46 μm) had no significant difference (P = 0.72).

**Table 2:** Comparison of choroidal and central macular thickness between diabetic and non-diabetic subjects


	**Diabetic cases**	**Non-diabetic cases**	**** ***P*** **-value**
Central choroidal thickness	247.80 ± 39.74	277.56 ± 34.15	0.001
Choroidal thickness at nasal point	238.63 ± 37.00	262.92 ± 34.44	0.004
Choroidal thickness at temporal point	239.30 ± 37.32	266.32 ± 32.47	0.001
Central macular thickness	251.51 ± 26.22	246.24 ± 22.46	0.72
	
	

Choroidal thickness in diabetic patients in group 1 had no significant difference with non-diabetic patients (P = 0.093, P = 0.379, and P = 0.450 for central, nasal, and temporal points, respectively) and diabetic patients in group 2 (P = 0.276, P = 0.830, and P = 0.465 for central, nasal, and temporal points, respectively) [Table 3]. However, choroidal thickness in diabetic patients in group 1 was significantly greater than patients in group 3 (P = 0.016, P = 0.042, and P = 0.031 for central, nasal, and temporal points, respectively). Choroidal thicknesses in diabetic patients in group 2 was significantly more than patients in group 3 (P = 0.003, P = 0.026, and P = 0.032 for central, nasal, and temporal points, respectively). Choroidal thicknesses was significantly greater in non-diabetic eyes than diabetic eyes in group 2 (P = 0.043, P = 0.025 and P = 0.0.016 for central, nasal, and temporal points, respectively) and group 3 (P = 0.001, P = 0.018 and P = 0.004 for central, nasal, and temporal points, respectively).

**Table 3:** The mean choroidal and central macular thickness in all study groups


	**Central choroidal thickness**	**Choroidal thickness at nasal point**	**Choroidal thickness at temporal point**	**Central macular thickness**
Group1	259.46 ± 39.73	246.12 ± 43.55	251.00 ± 42.91	258.29 ± 31.29
Group2	248.34 ± 33.72	237.55 ± 31.67	236.45 ± 33.95	254.17 ± 17.84
Group3	239.81 ± 43.08	234.62 ± 36.58	233.94 ± 35.27	245.03 ± 27.34
Group4	277.56 ± 34.16	262.92 ± 34.45	266.32 ± 32.48	246.24 ± 8.46
	
	

The mean central choroidal thickness in diabetic patients had no significant correlation with FBS (r = -0.063 and P = 0.685), duration of diabetes (r = -0.092 and P = 0.390), and age (r = 0.122 and P = 0.253).

Since patients with DME were excluded from this study, the mean central macular thickness was not different among all study groups.

##  DISCUSSION

In this study, we found that the choroidal thickness in non-diabetic patients was significantly greater than in diabetic patients. Moreover, this study showed that the choroidal thickness in non-diabetic patients was similar to that in diabetic patients with good glycemic control (HbA1c ≤ 7%) and was significantly greater than that in diabetic patients with moderate or poor glycemic control (HbA1c > 7%).

Lee and co-workers evaluated the choroidal thickness in diabetic patients using SD-OCT.^[11]^ They examined 203 eyes of 203 diabetic patients and 48 eyes of 48 non-diabetic controls and showed that the sub-foveal choroidal thickness was less in the eyes with NPDR or PDR than that in the healthy non-diabetic eyes; however, there was no significant difference between the diabetic eyes with no DR and the normal control eyes. Sudhalkar et al assessed choroidal thickness changes in different types of DR.^[15]^ They showed that diabetic patients with or without DR had a significantly thinner choroid compared to non-diabetic control group. Moreover, they found that patients with PDR had a thinner sub-foveal choroid than patients with NPDR. The authors concluded that choroidal thinning continued with increasing stages of DR.

In the current study, we showed that choroidal thickness in diabetic eyes is less than that in non-diabetic control eyes. This finding is in agreement with Lee et al,^[11]^ Sudhalkar et al,^[15]^ as well as some other previous studies reporting choroidal thinning in diabetic patients compared to non-diabetic patients.^[16,17]^ However, some other studies reported no significant difference in choroidal thickness between the diabetic and non-diabetic eyes.^[18]^

A previous study showed that abnormalities in the choriocapillaris and reduction of choroidal blood flow occurred in the early stages of DR,^[19]^ which may lead to choroidal hypoxia and ischemia and finally choroidal thinning. Yazici and co-workers compared the choroidal thickness in diabetic patients with and without polyneuropathy and healthy control subjects.^[20]^ They reported that the central choroid is thicker in diabetic patients compared to normal subjects and also showed that there was additional choroidal thickening in diabetic patients with polyneuropathy. They concluded that choroidal thickening may be related to autonomic dysregulation secondary to diabetic neuropathy. Kocasarac et al compared the choroidal thickness between diabetic patients with nephropathy and diabetic patients without nephropathy and found that the choroid is thinner in patients with diabetic nephropathy.^[21]^ Additionally, Faries et al reported that in diabetic patients with microalbuminuria, central choroidal thickness is less than diabetic patients without microalbuminuria.^[22]^ Microalbuminuria is an indicator of generalized vascular dysfunction;^[21]^ therefore, choroidal thinning in these patients may be a sign of choroidal vascular damage and dysfunction.

Sahinoglu-Keskek et al classified 122 eyes of 122 diabetic patients into three study groups including diabetic patients without DR, diabetic patients with DR and no macular edema, and diabetic patients with DR and macular edema.^[23]^ They reported that the sub-foveal choroid in patients with no DR was significantly thicker than that in patients with DR and without macular edema. Moreover, they reported that no significant differences in HbA1c levels were detected among the three study groups and concluded that there was no correlation between sub-foveal choroidal thickness and HbA1c values. Unlike Sahinoghlu-Keskek et al's study, we classified diabetic patients with the same DR severity based on HbA1c values and compared the choroidal thickness among them and found that the choroidal thickness in the healthy control group was nearly equal to that in patients with good glycemic control (HbA1c ≤ 7%) and was significantly more than the choroidal thickness in diabetic patients with moderate or poor glycemic control (HbA1c > 7%). Permanent high blood sugar levels in diabetic patients with inadequate treatment may lead to choroidal vascular damage and cause choroidal thinning in patient with DR.^[18]^ Therefore, in patients with uncontrolled DM and high levels of HbA1c, the choroid is expected to be thinner. Unsal et al reported a weak to moderate negative correlation between the sub-foveal choroidal thickness and HbA1c values, which is compatible with our results.^[24]^

Poorly controlled HbA1c is associated with more severe stages of DR;^[25]^ this may be due to the lower choroidal blood flow and choroidal hypoxia in the higher concentrations of blood sugar that ultimately may lead to choroidal thinning. Diabetic choroidopathy may play a major role in the pathogenesis of DR because the outer retinal layers are dependent on the choroid for nutrition and oxygenation.^[7]^

In the current study, we found that there was no significant correlation between the duration of diabetes and choroidal thickness. This is compatible with the studies of Sahinoghlu-Keskek et al and Shen et al.^[23,26]^

We are aware of some of the limitations of our study. First, we had no axial length data; however, the patients with high refractive errors were excluded. The possibility of measurement errors due to manual measurement and measurement by a single observer constitute other limitations.

In conclusion, we showed that the choroidal thickness in normal control subjects was almost equal to diabetic patients with good glycemic control (HbA1c ≤ 7%) and was significantly greater than choroidal thickness in diabetic patients with moderate or poor glycemic control (HbA1c > 7%). Therefore, better diabetic control with HbA1c ≤ 7% may prevent choroidal vascular damage and choroidal thinning and finally prevent the development of DR. However, future studies with larger sample size are required to establish the correlation of choroidal thickness with HbA1c and to determine the optimal cutoff value of HbA1c that affects the choroidal vascular system.

##  Financial Support and Sponsorship

Nil.

##  Conflicts of Interest

There are no conflicts of interest.
